# Lacking social support is associated with structural divergences in hippocampus–default network co-variation patterns

**DOI:** 10.1093/scan/nsac006

**Published:** 2022-01-27

**Authors:** Chris Zajner, R Nathan Spreng, Danilo Bzdok

**Affiliations:** McConnell Brain Imaging Centre (BIC), Montreal Neurological Institute (MNI), Faculty of Medicine, McGill University, Montreal H3A2B4, Canada; McConnell Brain Imaging Centre (BIC), Montreal Neurological Institute (MNI), Faculty of Medicine, McGill University, Montreal H3A2B4, Canada; McConnell Brain Imaging Centre (BIC), Montreal Neurological Institute (MNI), Faculty of Medicine, McGill University, Montreal H3A2B4, Canada; Department of Biomedical Engineering, Faculty of Medicine, McGill University, Montreal H3A2B4, Canada; Mila—Quebec Artificial Intelligence Institute, Montreal H2S 3H1, Canada

**Keywords:** social isolation, population neuroscience, hippocampus subfields, default network fragmentation, higher-order association cortex

## Abstract

Elaborate social interaction is a pivotal asset of the human species. The complexity of people’s social lives may constitute the dominating factor in the vibrancy of many individuals’ environment. The neural substrates linked to social cognition thus appear especially susceptible when people endure periods of social isolation: here, we zoom in on the systematic inter-relationships between two such neural substrates, the allocortical hippocampus (HC) and the neocortical default network (DN). Previous human social neuroscience studies have focused on the DN, while HC subfields have been studied in most detail in rodents and monkeys. To bring into contact these two separate research streams, we directly quantified how DN subregions are coherently co-expressed with specific HC subfields in the context of social isolation. A two-pronged decomposition of structural brain scans from ∼40 000 UK Biobank participants linked lack of social support to mostly lateral subregions in the DN patterns. This lateral DN association co-occurred with HC patterns that implicated especially subiculum, presubiculum, CA2, CA3 and dentate gyrus. Overall, the subregion divergences within spatially overlapping signatures of HC–DN co-variation followed a clear segregation into the left and right brain hemispheres. Separable regimes of structural HC–DN co-variation also showed distinct associations with the genetic predisposition for lacking social support at the population level.

## Introduction

Our brains are highly attuned to mediating social relationships. Yet, this unique property may make us especially vulnerable in times of social isolation. The hippocampus (HC) and the highly associative default network (DN) in particular play key roles in representing and integrating knowledge about the panoply of people’s social worlds (Spreng and Andrews-Hanna, 2015; Tavares *et al.*, 2015). Evidence across the primate lineage demonstrates expansion of the brain toward continuously larger association cortex volumes. It has been suggested that this selective expansion of association cortex brain regions has especially coincided with the benefits of social bonding and coping with life in social groups (Dunbar, 1998; Dunbar and Shultz, 2007). Additionally, based on within-species assessments among humans, the size of one’s social network has been found to correlate with the structure of brain regions, including key parts of the DN (Lewis *et al.*, 2011). This finding receives further support from monkey experiments aimed at controlling group size (Sallet *et al.*, 2011). In this light, recently expanded regions of the association cortex appear to be related to the advanced processing capacities required for navigating social exchange with others (Schurz *et al.*, 2021a).

However, the neocortical nodes of the DN have long been emphasized in the brain-imaging community to be integral for internally generated or self-directed cognitive functions, as opposed to externally or environment-oriented processing (Andrews-Hanna *et al.*, 2014). This raises the question of how the DN supports a central role in social embeddedness. Emotional connection is one fundamental aspect of social cognition. Yet, this domain is classically believed to be subserved by the limbic system, which is often considered to include the HC (Schurz *et al.*, 2021b). Although controversy surrounds the roles and anatomical composition of the limbic system, as well as the value of the concept itself (Ledoux, 1991; Rolls, 2015), it traditionally denotes brain regions associated with emotion (LeDoux, 2000). On the other hand, the more cognitive and abstract reasoning-based aspects of social processing may be preferentially subserved by neocortical regions of the DN, such as the medial prefrontal cortex (mPFC), inferior parietal lobule (IPL) and temporoparietal junction (TPJ). For example, these same regions are associated with general perspective-taking competence (Lewis *et al.*, 2011) and show neural activity responses when thinking about others (Krienen *et al.*, 2010). Thus, the relationship between the internal representation of the social world and DN regions could perhaps be a key factor behind the recent discovery that the DN is the network circuit with the strongest links to subjective social isolation (Spreng *et al.*, 2020). Yet, we still have an incomplete understanding of how social isolation and the disparate subsystems within the higher association cortex are inter-linked with their affiliates in the allocortex.

This knowledge gap is in part due to inherent methodological challenges posed by the endeavor of studying DN regions that are particularly evolved in the human species. From a comparative perspective, progress in elucidating the DN in humans is hampered by the difficulty of confidently matching distinct DN subregions to counterparts in the brains of our monkey ancestors or even other animals. Indeed, several neocortical areas of the human association cortex may have no definitive homolog in non-human animals (Petrides *et al.*, 2012). For example, there are hurdles to the attempts of identifying homologous structures of the human TPJ and mPFC in the monkey brain (Saxe, 2006; Mars *et al.*, 2013). Overall, such incongruences in comparative studies have impeded ‘the ability to compare experimental findings from non-human primates with results obtained in functional and structural neuroimaging of the human brain’ (Petrides *et al.*, 2012).

In contrast, there is an extensive knowledge base on the evolutionarily more conserved HC in the allocortex (Buzsaki, 2006; Xu *et al.*, 2020). This is due to the ready possibility for conducting direct experiments on the HC of animals, which are typically infeasible in humans. For example, invasive studies in living animals using direct axonal tracing, gene expression probes, optogenetic manipulation and single-cell electrophysiological recording in the rodent and monkey HC have enriched our understanding of this structure and its functionally specialized subfields. There is also accumulating knowledge of the effects of social isolation on specific subregions of the animal HC (Kogan *et al.*, 2000; Silva-Gomez *et al.*, 2003; Ibi *et al.*, 2008; Biggio *et al.*, 2019). For these reasons, the neuroscientific understanding of the neocortical subregions of the DN remains more opaque than those of the HC. Thus, simultaneously investigating these two functionally interacting neural systems opens a window, which can eventually allow illuminating key properties of human DN subregions through the lens of their partner HC subregions.

We expected concordances in how these allocortical and neocortical neural systems are related to social isolation. This is because both circuits are implicated in serving social interaction, such as the abstract representation of other people’s purview of the world. On the one hand, the DN is well-known to be involved in both representing information about other people (Courtney and Meyer, 2020) and taking other peoples’ perspectives (Saxe and Kanwisher, 2003; Frith and Frith, 2006; Jamali *et al.*, 2021; Numssen *et al.*, 2021). On the other hand, the HC of various animal species has been shown to serve proto-forms of such functions (Danjo *et al.*, 2018; Omer *et al.*, 2018; Oliva *et al.*, 2020). This idea is supported by the wide range of information domains, which individual HC neurons are reported to be capable of representing, as evidenced by invasive electrophysiological recordings in rodents and monkeys (Eichenbaum *et al.*, 1999; Behrens *et al.*, 2018; Bellmund *et al.*, 2018). These include spatial boundaries (Lever *et al.*, 2009), head direction (Taube *et al.*, 1990), goal direction (Sarel *et al.*, 2017), sound frequency (Aronov *et al.*, 2017), odor (Wood *et al.*, 2000; Radvansky and Dombeck, 2018), time (MacDonald *et al.*, 2011) and reward (Gauthier and Tank, 2018). These representations related to single-cell activity extend to social information as well. For example, in rodents and bats, individual HC neurons have been shown to specifically represent the location of peers within a spatial environment (Danjo *et al.*, 2018; Omer *et al.*, 2018; Oliva *et al.*, 2020).

The human HC is also increasingly believed to represent discrete items of social information. For example, *invivo* electrophysiological experiments in epilepsy patients have shown that single HC neurons in the medial temporal lobe consistently respond to pictures of the same person from diverse viewpoints (Quiroga *et al.*, 2005, 2009; Rey *et al.*, 2020). Additionally, a similar study of neurosurgical patients found that single HC neurons tend to respond to different images of people if those images were previously judged by the patient to be similar (De Falco *et al.*, 2016). The conjunction of these earlier studies suggests that neurons in the HC of human and non-human mammals play a fundamental role in recognizing and representing specific peers. These social representations may hence be embedded within neural representations of ‘social spaces’ mediated by the entorhinal cortex.

Indeed, the entorhinal cortex—which is the main input and output structure of the HC—has repeatedly been shown to code an animal’s location through an ensemble of ‘grid cells’ (Hafting *et al.*, 2005; Moser *et al.*, 2008; Jacobs *et al.*, 2013). These dedicated neurons are believed to discharge according to fields that tessellate the environment with regular hexagonal patterns. In fact, such ‘grid cells’ have been demonstrated to map multiple different domains of information, such as space (Hafting *et al.*, 2005; Moser *et al.*, 2008; Jacobs *et al.*, 2013), time (Kraus *et al.*, 2015) and speed (Kropff *et al.*, 2015). Some studies have also shown that the presubiculum (PrS) and parasubiculum support such ‘grid cell’ representations (Boccara *et al.*, 2010).

The constant representation of spatial environments by grid cells has been further suggested as a bedrock for a long discussed role of the HC—to instantiate ‘cognitive maps’, classically maps of space (Tolman, 1948; O’keefe and Nadel, 1978). Later, it was suggested that this function in spatial conceptualization has been co-opted in the primate brain to help instantiate other abstract maps of related entities (Constantinescu *et al.*, 2016; Behrens *et al.*, 2018; Bellmund *et al.*, 2018). Recent evidence suggests that similar spatial maps are also represented in the orbital frontal cortex region of the DN (Wikenheiser *et al.*, 2021). Evidence of a cognitive map of interpersonal ties has been further associated with neural activity responses of the human HC. This involved both social agent ‘nodes’ and their social relationship ‘edges’ (Tavares *et al.*, 2015). In particular, hippocampal functional magnetic resonance imaging (fMRI) activity could track the position of characters within a social hierarchy as indexed by ‘power’ and ‘affiliation’ (Tavares *et al.*, 2015). Moreover, in this human fMRI experiment, individuals with better social skills showed more pronounced fMRI activity responses (Tavares *et al.*, 2015). It is thus conceivable that advanced types of social cognition, such as perspective-taking, require accessing and binding information within an abstract social relationship ‘map’ mediated by the HC. If so, we reasoned that the hippocampal subregions that play central roles in instantiating a cognitive map are potentially linked to the regular exchange in one’s wider social networks and lack thereof. The shared functions of the HC and DN thus point toward principled co-variation between their subcomponents.

In the past, brain-imaging studies aiming at brain parcellation have typically investigated either the DN (e.g. Schurz *et al.*, 2014) or the HC alone (e.g. Plachti *et al.*, 2019). Although there is extensive research from animal studies on anatomically defined subregions of the HC, the extension of this work to investigations on the human HC is still lacking. Advances in the automatic segmentation of the HC using *ex vivo* brain imaging (Iglesias *et al.*, 2015; Wisse *et al.*, 2017) now allow reliable assessments of microanatomically defined HC subregions in a way that scales to the ∼40 000 UK Biobank Imaging cohort. This enables deeper analyses of the principled inter-relationships between the evolutionarily more conserved allocortical HC and DN of the recently expanded neocortex. By leveraging a framework for high-dimensional decomposition at a fine-grained subregion resolution, we here uncover principled co-variation signatures that delineate how HC subregion volumes are co-expressed with DN subregion volumes. We also detail the structural deviations of these co-variation signatures that characterize objective social isolation. Moreover, enabled by the availability of genetic data, we investigate how these structural brain patterns are associated with the genetic predisposition to experience social isolation. This is accomplished by examining relationships between the expression of HC–DN co-variation across individuals and the genetic liability of social isolation based on a polygenic risk score (PRS) model. Robust links between these two sources of variation—in structural brain morphology and purely genetic predisposition—may thus speak to how the heritable components of social support are linked to the aspects of brain morphology examined here at the population level.

## Material and methods

### Data resources

The UK Biobank is a prospective epidemiology resource that offers extensive behavioral and demographic assessments, medical and cognitive measures, as well as biological samples in a cohort of ∼500 000 participants recruited from across Great Britain (https://www.ukbiobank.ac.uk/). This openly accessible population dataset aims to provide brain imaging for ∼100 000 individuals planned for completion in 2022. The present study was based on the recent data release from February/March 2020. To improve comparability and reproducibility, our study built on the uniform data preprocessing pipelines designed and carried out by FMRIB, Oxford University, UK (Alfaro-Almagro *et al.*, 2018). Our study involved data from 38 701 participants with brain-imaging measures and expert-curated image-derived phenotypes of gray matter (GM) morphology (T1-weighted MRI) from 48% men and 52% women, aged 40–69 years when recruited (mean age 55 years, s.d. 7.5 years). The present analyses were conducted under UK Biobank application number 25163. All participants provided informed consent. Further information on the consent procedure can be found elsewhere (http://biobank.ctsu.ox.ac.uk/crystal/field.cgi?id=200).

### Target phenotype for objective social isolation

To capture an objective measure of the frequency of social interactions, our UK Biobank analyses were based on answers to the question ‘How often are you able to confide in someone close to you?’(data field 2110). Our study modeled lack of social support as less than ‘daily or almost daily’ (yes or positive answer) against confiding in others more often (treated as no or negative answer).

Measures of social relationship quality represent a widely accepted and widely investigated component of social embeddedness (Cohen and Hoberman, 1983; Hawkley *et al.*, 2005). Lack of social support is commonly viewed as an objective measure of weak social connection with other people. For example, the Social Relationships scales are part of the NIH Toolbox (Cyranowski *et al.*, 2013) feature dimensions of social networks, which closely resembles our measure of social support. In general, a variety of studies showed single-item measures of social isolation traits to be reliable and valid (Mashek *et al.*, 2007; Dollinger and Malmquist, 2009; Atroszko *et al.*, 2015). The sociodemographic differences between low and high social support individuals in the UK Biobank have been reported elsewhere (Schurz *et al.*, 2021b), but we also show this data in Supplementary Table S2.

### Brain-imaging and preprocessing procedures

MRI scanners (3T Siemens Skyra) were matched at several dedicated data collection sites with the same acquisition protocols and standard Siemens 32-channel radiofrequency receiver head coils. To protect the anonymity of the study participants, brain-imaging data were defaced and any sensitive meta-information was removed. Automated processing and quality control pipelines were deployed (Miller *et al.*, 2016; Alfaro-Almagro *et al.*, 2018). To improve the homogeneity of the imaging data, noise was removed by means of 190 sensitivity features. This approach allowed for the reliable identification and exclusion of problematic brain scans, such as due to excessive head motion.

The structural MRI data were acquired as high-resolution T1-weighted images of brain anatomy using a three-dimensional MPRAGE sequence at 1 mm isotropic resolution. Preprocessing included gradient distortion correction, field of view reduction using the Brain Extraction Tool (Smith 2002) and FLIRT (Jenkinson and Smith 2001; Jenkinson *et al*. 2002), as well as non-linear registration to MNI152 standard space at 1 mm resolution using FNIRT (Andersson *et al*. 2007). To avoid unnecessary interpolation, all image transformations were estimated, combined and applied by a single interpolation step. Tissue-type segmentation into cerebrospinal fluid, GM and white matter (WM) was applied using FAST (FMRIB’s Automated Segmentation Tool, Zhang *et al*. 2001) to generate full bias-field-corrected images. SIENAX (Smith *et al*. 2002), in turn, was used to derive volumetric measures normalized for head size.

For the DN, volume extraction was anatomically guided by the Schaefer–Yeo reference atlas (Schaefer *et al.*, 2017). Among the total 400 parcels, 91 subregion definitions are provided as belonging to the DN among the 7 canonical networks. For the HC, 38 volume measures were extracted using the automatic Freesurfer sub-segmentation (Iglesias *et al.*, 2015). The allocortical volumetric segmentation draws on a probabilistic HC atlas with ultra-high resolution at ∼0.1 mm isotropic. This tool from the Freesurfer 7.0 suite gives special attention to surrounding anatomical structures to refine the HC subregion segmentation in each participant. The automatically derived HC sub-segmentation used in the UK Biobank is indeed at the forefront of imaging parcellation and involved development by imaging experts as well as a Bayesian inference approach with ultra-high resolution *ex vivo* data from autopsy brains. This segmentation protocol has additionally been shown to provide robustly distinguishable details at the imaging and genetic levels (Foo *et al.*, 2021). For example, it has been found using this subregion atlas that certain hippocampal subregions had stronger associations with age, sex and PRS for Alzheimer’s disease (AD). More specifically, these investigators sought to elucidate whether the volume of hippocampal subregions could serve as a biomarker of risk for AD in a large population dataset. Overall, these researchers show that this subregion atlas reveals that certain hippocampal subregions had stronger associations with age, sex and PRS for AD. More specifically, they found a particular association between PRS for AD and lower volume in left CA1, left ML, left GC-DG-ML, left, CA4, right subiculum (Sub), bilateral tail and bilateral hippocampal amygdala transition area (HATA). In this and similar UK Biobank studies, such automatic HC parcellations have been found to provide reliable associations between genetic markers and specific hippocampal subregions (Van der Meer *et al.*, 2020).

As a preliminary data-cleaning step, building on previous UK Biobank research (Spreng *et al.*, 2020; Schurz *et al.*, 2021b), inter-individual variation in brain region volumes that could be explained by nuisance variables of no interest was regressed out: body mass index, head size, head motion during task-related brain scans, head motion during task-unrelated brain scans, head position and receiver coil in the scanner (*x*, *y* and *z*), position of scanner table, as well as the data acquisition site, in addition to age, age^2^, sex, sex*age and sex*age^2^. The cleaned volumetric measures from the 91 DN subregions in the neocortex and the 38 HC subregions in the allocortex served as the basis for all subsequent analysis steps.

### Analysis of co-variation between HC subregions and DN subregions

As the keystone of the analytical workflow, we sought dominant regimes of structural correspondence—signatures or ‘modes’ of population co-variation that provide insights into how structural variation among the segregated HC can explain structural variation among the segregated DN (see Figure 6). Canonical correlation analysis (CCA) was a natural choice of method to interrogate such multivariate inter-relations between two high-dimensional variable sets (Witten *et al.*, 2009; Bzdok *et al.*, 2019; Wang *et al.*, 2020).

A first variable set $X$ was constructed from the DN subregion volumes (number of participants × 91 DN parcels matrix). A second variable set $Y$ was constructed from the HC subregion volumes (number of participants × 38 HC parcels matrix):

$$X \in {\mathbb{R}^{n \times p}}$$

$$Y \in {\mathbb{R}^{n \times q}}$$

where $ n$ denotes the number of observations or participants, $p$ is the number of DN subregions and $q$ is the number of HC subregions. Each column of the two data matrices was *z*-scored to zero mean (i.e. centering) and unit variance (i.e. rescaling). CCA addresses the problem of maximizing the linear correlation between low-rank projections from the two variable sets or data matrices. The two sets of linear combinations of the original variables are obtained by optimizing the following target function:

$${L_X} = XV {L_Y} = YU$$

$${l_{X,l}} = X{v_l} {l_{Y,l}} = Y{u_l}$$

$$corr\left( {{l_{X,l}},{l_{Y,l}}} \right) \propto l_{X,l}^T{l_{Y,l}} = max$$

where $V$ and $U$ denote the respective contributions of $X$ and $Y$,$ {L_X}$ and ${L_X}$ denote the respective latent ‘modes’ expression of joint variation (i.e. canonical variates) based on patterns derived from $X$ and patterns derived from $Y$, ${l_{X,l}}$ is the $l$th column of ${L_X}$ and ${l_{Y,l}}$ is the $l$th column of ${L_Y}$. We define mode as general principles of population variation in our target neural circuits that can be reliably extracted in brain structure at the population level. Further, a co-variation *signature* henceforth refers to a mode overall (including its two corresponding canonical vectors), whereas a *pattern* will refer to a mode’s constituent canonical vectors for the corresponding HC and DMN subregion sets. The goal of our CCA application was to find pairs of latent vectors ${l_{X,l}}$ and ${l_{Y,l}}$ with maximal correlation in the derived latent embedding. In an iterative process, the data matrices $X$ and $Y$ were decomposed into $L$ components, where $L$ denotes the number of modes given the model specification. In other words, CCA involves finding the canonical vectors ${u_{}}$ and ${v_{}}$ that maximize the (symmetric) relationship between a linear combination of DN volume expressions ($X$) and a linear combination of HC volume expressions ($Y$). CCA thus identifies the two concomitant projections $X{v_l}$ and $Y{u_l}$. These yielded the optimized co-occurrence between combined subregion variation inside the segregated DN and combined subregion variation inside the segregated HC across participants.

In other words, each estimated co-variation signature identified a constellation of within-DN volumetric variation and a constellation of within-HC volumetric variation that go hand-in-hand with each other. Namely, every co-variation signature is constituted by the relative structural relationships among the DN and HC subregion sets, as described by each respective mode. A pattern on the other hand refers to the relative contributions (weights) of each subregion within a canonical vector, which is a result of the analytic workflow (CCA step). The set of $k$ orthogonal modes of population co-variation are mutually uncorrelated by construction (Wang *et al.*, 2020). They are also naturally ordered from the most important to the least important HC–DN co-variation mode based on the amount of variance explained between the allocortical and neocortical variable sets (see Supplementary Figure S1 for HC, see Supplementary Figure S2 for DN). The first and strongest mode therefore explained the largest fraction of joint variation between combinations of HC subregions and combinations of DN subregions. Each ensuing cross-correlation signature captured a fraction of structural variation that is not explained by one of the $k - 1$ other modes. The variable sets were entered into CCA after a confound-removal procedure based on previous UK Biobank research (cf. above).

### Group difference analysis

**Fig. 1.**
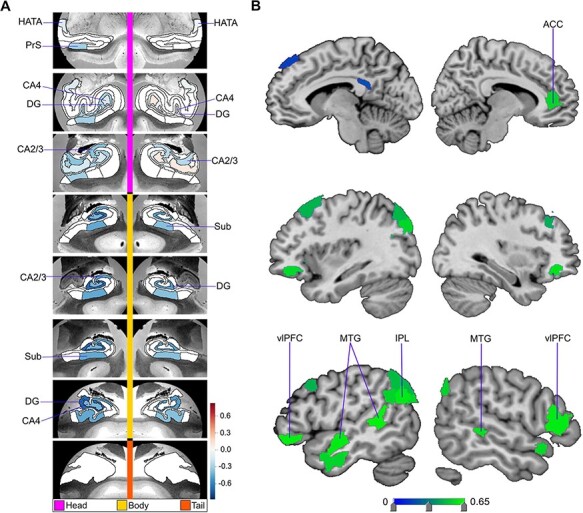
Low social support is associated with divergences in distinct subregions in the dominant signature of HC–DN co-variation. In ∼40 000 UK Biobank participants, we explored the structural co-variation between the 38 allocortical subregions of the HC and 91 neocortical subregions of the DN by means of a two-view decomposition involving CCA. We subsequently determined how the ensuing subregion patterns diverged in individuals with lack of social support (bootstrap difference test, cf. ‘Materials and methods’ section). The leading mode of the obtained CCA solution achieves the most explanatory co-variation, with a canonical correlation of rho = 0.51. (A) The figure shows the HC subregion patterns (left, one canonical vector of mode 1) with parameter weights that robustly diverge between low and high social support groups; mapped onto eight consecutive coronal slices of the left and right HC in the anterior (top) to posterior (bottom) direction. (B) The figure shows the DN subregions patterns (right, other canonical vector of mode 1) that robustly diverge between the low and high social support groups. Although the analyses of the HC and DN are obtained from an integrated framework, different color schemes are used for the HC and DN visualizations to indicate the difference in the anatomical scales between the macroscopic DN and microanatomically defined HC subregions. Overall, within the leading signature of structural co-variation between the HC and the DN, there are specific subregions whose volumes systematically diverge in individuals with low social support. vlPFC = ventrolateral prefrontal cortexDG = GC-DG-ML.

For each of the derived population modes of HC–DN co-variation, we then performed a rigorous group contrast analysis for social isolation. We aimed to identify which anatomical subregions show statistically defensible deviation in the socially isolated group compared to the control group. For the low social support trait, we carried out a principled test for whether the solution vector obtained from CCA (i.e. canonical vectors, cf. above) in the socially isolated group is systematically different from the solution vector in the control group. This analysis step elucidated the HC and DN subregions that showed structural divergences in the particular context of our target phenotype, namely lacking social support (see Figure 1).

More specifically, following previous UK Biobank research (Schurz *et al.*, 2021b), we carried out a bootstrap difference test of the CCA solution from the socially isolated group *vs* socially well-connected group (Efron and Tibshirani, 1993). In 100 bootstrap iterations, we randomly pulled participant samples with replacement to build an alternative dataset (with the same sample size) that we could have gotten. We subsequently performed CCA in parallel by fitting one separate model to each of the two groups. In each resampling iteration, this approach thus carried out a separate estimation of the doubly multivariate correspondence between HC subregions and DN subregions in each particular group. The two distinct CCA solutions from each iteration were then matched mode-by-mode regarding sign invariance and mode order. Canonical vectors of a given mode that carried opposite signs were aligned by multiplying one with −1. The order of the CCA modes was aligned based on pairwise Pearson’s correlation coefficient between the canonical vectors from each estimated CCA model. After mode matching, we directly estimated the resample-to-resample effects by elementwise subtraction of the corresponding canonical vectors of a given mode *k* between the two groups. We finally recorded these difference estimates for each vector entry (each corresponding to the degree of deviation in one particular anatomical subregion). The subregion-wise differences were ultimately aggregated across the 100 bootstrap datasets to obtain a non-parametric distribution of group contrast estimates.

We thus propagated the variability attributable to participant sampling into the computed uncertainty estimates of group differences in the UK Biobank population cohort. Statistically relevant alteration of anatomical subregions in social isolation was determined by whether the two-sided confidence interval included zero or not according to the 10/90% bootstrap-derived distribution of difference estimates (Schurz *et al.*, 2021b). Remaining faithful to our multivariate analytical strategy and research question, this non-parametric approach directly quantified the statistical uncertainty of how social isolation is manifested in specific subregions of the HC–DN axis.

### Analysis of how individual expressions of HC–DN co-variation are linked to the genetic predisposition for social isolation

PRS is a genome-wide analysis technique that has been shown to successfully quantify the genetic predisposition of individuals for a variety of phenotypes. The approach has become especially potent for complex phenotypes that implicate tens of thousands of common-variant single-nucleotide polymorphisms (SNPs) with individually small-effect sizes, such as major psychiatric diseases (HLA-C, H., 2009; Kuchenbaecker *et al.*, 2017; Inouye *et al.*, 2018; Khera *et al.*, 2018; Elliott *et al.*, 2020). PRS has is also considered to be a sharp tool for heritability analyses due to the advent of large population datasets (e.g. the UK Biobank) (Choi *et al.*, 2020; Wray *et al.*, 2021). Such data resources have allowed the investigation of the relationship between SNP-based genetic variation and inter-individual differences in a particular phenotype, which includes neuroimaging-derived phenotypes (Elliott *et al.*, 2018; Smith *et al.*, 2021). For the purpose of the present study, we have constructed a PRS model for the low social support trait. The subject-specific risk scores were then regressed onto our expressions of HC–DN modes (i.e. canonical variates). Our integrative imaging-genetics approach aimed to disentangle which mode expressions have reliable links to the genetic vulnerability to low social support trait as attributable to thousands of genetic variants.

As is common for PRS analysis workflows, summary statistics from previously conducted genome-wide association studies (GWAS) on our target phenotypes were used as the backdrop to determine how several hundred thousand SNPs are associated with the low social support trait. The summary statistics for low social support were obtained from a GWAS that was conducted as part of the Canadian Longitudinal Study on Aging. Quality control was implemented by excluding SNPs with a minor allele frequency of <1%, as well as excluding SNPs with imputation information score of <0.8. Mismatching, duplicate and ambiguous SNPs were also disregarded from further analysis. Quality control on the base data also involved excluding individuals with a difference between reported sex and that indicated by their sex chromosomes and removing overlapping samples.

The quality-controlled summary statistics were used as starting point for the PRS model that was built and applied using the PRSice framework (http://www.prsice.info). This software tool uses the available collection of effect sizes of candidate SNPs to form single-subject predictions of genetic predisposition for a phenotype of interest. More specifically, this tool determined the optimal PRS model based on the UK Biobank participants (training data, *n* = 253 295) of European ancestry who did *not* provide any brain-imaging data (at the time of study). This model training step involved automated adjustments, such as identifying ideal clumping and pruning choices, to select the thresholds that decide which SNPs are included in the PRS model. Subsequently, once optimized, the final PRS model was then used to predict the genetic predisposition for each of 23 423 UK Biobank participants of European ancestry *with* brain-imaging data (test data). This PRS model consisted in pooling across additive effects of weighted SNPs, whereby the weighted sum of the participants’ genotypes was computed as follows:

$$prs = \sum {{{\bf{g}}_{\rm{i}}}} *\,\,{\hat \beta _i}$$

where ${{\rm{g}}_{\rm{i}}}$ denotes an individual’s genotypes at SNP $i$ (values 0, 1, or 2), $ {\hat \beta _i} $ is the obtained point estimates of the per-allele effect sizes at SNP $i$ and $j$ is a particular individual (Choi *et al.*, 2020).

Finally, Bayesian linear regression was used to regress the subject-specific predictions of genetic liability for the low social support trait onto the participant expressions of HC–DN co-variation modes. To this end, the individuals in the top 5% predictions (i.e. highest PRS estimates) and the individuals in the bottom 5% predictions (i.e. lowest PRS estimates) were considered as a binary outcome in a Bayesian logistic regression model with participant-wise mode expressions serving as input variables (Lecarpentier *et al.*, 2017; Fan *et al.*, 2019; Meisner *et al.*, 2020). In this multiple regression setup, PRS for low social support was regressed against each of the 25 canonical variates (linearly uncorrelated by construction) on the HC side for every individual. An analogous multiple regression model was estimated for the (uncorrelated) 25 canonical variates from the DN side. The fully Bayesian model specification for these regression analyses was as follows:

$$\eqalign{{y_{prs}}={{\bf{x}}_1}*{\beta_{mod{e_1}}} +\ldots+{{\bf{x}}_{25}}*{\beta_{mod{e_{25}}}}+{\alpha _{men\left[ {sex}\right]}}+{\alpha _{women\left[{sex}\right]}}{\rm{ }} \\ \cr +{\alpha_{men\_age\left[{sex}\right]}}*ag{e_{men}}+{\alpha _{women\_age\left[{sex}\right]}}*ag{e_{women}}}$$

$$ {\beta_j} \sim {{\mathcal{N}}_{\rm{j}}}\left( {0,{\ }1} \right)$$

$$ {\alpha_{men}} \sim {\mathcal{N}}\left( {0,{\ }1} \right)$$

$$ {\alpha_{women}} \sim {\mathcal{N}}\left( {0,{\ }1} \right)$$

$$ {\alpha_{men\_age}} \sim {\mathcal{N}}\left( {0,{\ }1} \right)$$

$${\alpha_{women\_age}} \sim {\mathcal{N}}\left( {0,{\ }1} \right)$$

where ${\beta_j}$ denotes the slopes for the subject-specific 25 mode expressions as canonical variates ${{\rm{x}}_{\rm{j}}}$, and ${y_{prs}}$ denotes the PRS estimates of each participant. Potential confounding influences were acknowledged by the nuisance variables α, which accounted for variation that could be explained by sex and (*z*-scored) age. Once the Bayesian model solution was approximated using Markov chain Monte Carlo sampling, it yielded fully probabilistically specified posterior parameter distributions for each $\beta$ coefficient corresponding to one of the signatures of allocortical–neocortical co-variation (cf. above). The association with trait heritability of a mode expression was then determined based on how robustly their corresponding model coefficients deviated from 0 (e.g. >95% of model coefficient posterior probability excluded a value of 0).

## Results

### Structural co-variation between HC and DN at the subregion level

We explored the principal signatures of structural co-variation between the full set of 38 hippocampal subregions and the full set of 91 DN subregions. The concurrent patterns within subregion variation among the HC and those within the DN were computed using a doubly multivariate learning algorithm. In so doing, we achieved a co-decomposition of hippocampal subregion volumes and DN subregion volumes. Each of the top 25 modes of co-variation was characterized based on how much of joint variance a particular signature explained: with the most explanatory signature (mode 1) achieving a canonical correlation of rho = 0.51 (measured as Pearson’s correlation coefficient) (see Supplementary Table S1). The second most explanatory signature (mode 2) achieved a canonical correlation of rho = 0.42, the third signature rho = 0.39, the fourth signature rho = 0.31, the fifth signature rho = 0.27 and the sixth rho = 0.23; through to the 25th signature that had rho = 0.06 (see Supplementary Table S1 for full list). This analysis thus established the scaffold for all subsequent analyses that delineates how multiple complementary hippocampal patterns co-vary hand-in-hand with DN patterns across individuals.

### Differences in the HC–DN co-variation in social isolation

Based on the identified population signatures of HC–DN co-variation, we investigated the neurobiological manifestations of social isolation in our UK Biobank sample. This was accomplished by examining robust subregion-level divergences in how hippocampal patterns are co-expressed with DN patterns that characterize groups of socially isolated *vs* control participants (i.e. low *vs* high social support). To this end, we analyzed objective social isolation by a rigorous group difference analysis between the structural patterns of co-variation in the low social support and high social support groups. This approach revealed the precise subregions contributing to the structural HC–DN co-variation that systematically diverged between the two groups, for each mode of the CCA.

We uncovered a multitude of modes with systematic group differences in either specific HC or DN subregions (see Table 1). We also found modes with no significant structural divergences in any HC or DN subregion. From here on, a subregion that was observed to have a robustly different weighting within a modes canonical vector, between low and high social support groups, is termed a ‘hit’ (i.e. an observed structural divergence in individuals with low social support). Across all 25 examined modes, contrasting low *vs* high social support, we identified hits in 32 HC subregions and 50 hits in DN subregions. Most of these hits occurred in earlier modes, with all the hits occurring between modes 1 and 7. Just in the first three modes, we found 24 HC subregion hits (70.6% of the HC total). In the first mode alone, we observed 26 DN hits (52% of the DN total). Across all modes, the largest number of HC hits were observed in PrS (5 hits), Sub (5), CA2/3 head (4) and GC-DG-ML (4). Regarding the parallel DN divergences, we found the largest number of hits in lateral cortical subregions (78% of the total), such as the middle temporal gyrus (MTG), temporal pole and IPL, with less number of hits in midline subregions. There was a total number of hits in 17 temporal (34%), 17 prefrontal (34%), 11 parietal (22%) and 5 posterior cingulate (10%) subregions.

**Table 1.** Hippocampus subregion divergences across hippocampus–default network co-variation modes

Mode	CA1	CA2/3	CA4	DG	HATA	Para	PrS	Sub	Fissure	Fimbria	ML	Tail
1	0	3	3	4	2	0	1	2	0	0	0	0
2	0	0	0	0	0	2	2	1	0	0	0	2
3	0	2	0	0	0	0	0	1	0	0	0	0
4	0	0	0	0	1	0	1	0	0	1	0	0
5	0	0	0	0	0	0	1	1	0	1	1	0
6	0	0	0	0	0	0	0	0	0	0	0	0
7	0	0	0	0	0	1	0	0	0	0	0	0
Total	0	5	3	4	3	3	4	5	0	2	1	2

There were no subregion divergences between modes 8 and 25.

**Fig. 2.**
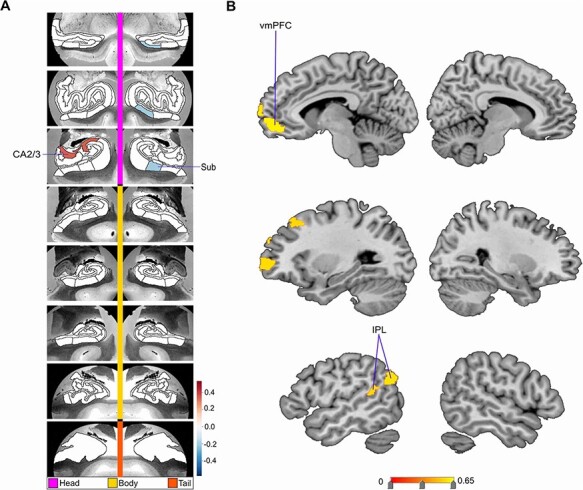
Divergences in CA2/3, Sub and left DN subregions are associated with low social support. Mode 3 of the CCA solution achieves the third most explanatory HC–DN co-variation, with a canonical correlation of rho = 0.39. (A) The figure shows the HC subregion patterns (left, one canonical vector of mode 3) with parameter weights that robustly diverge between low and high social support groups—mapped onto eight consecutive coronal slices of the left and right HC in the anterior (top) to posterior (bottom) direction. (B) The figure shows the DN subregion patterns (right, other canonical vector of mode 3) that robustly diverge between the low and high social support groups. Similar to mode 1 (see Figure 1), within the third most explanatory mode, low social support has preferential effects on CA2/3 and Sub of the HC, as well as left hemisphere DN subregions.

The divergences between the low versus high social support groups for mode 1 (see Figure 2) revealed a rough synopsis of the hit locations for the totality of the observed modes. In the dominant mode, we observed hits in bilateral CA2/3 head and body, bilateral CA4 head, left CA4 body, bilateral HATA, left PrS head, bilateral Sub body and bilateral GC-DG-ML head and body, with 26 hits in DN subregions (8 temporal, 7 parietal, 10 prefrontal and 1 posterior cingulate). Additionally, the HC subregion hits with the strongest weights included left CA2/3 body and bilateral GC-DG-ML. On the flipside of our model, the DN subregion hits with the strongest weights were bilateral prefrontal cortex, left MTG and left IPL.

**Fig. 3.**
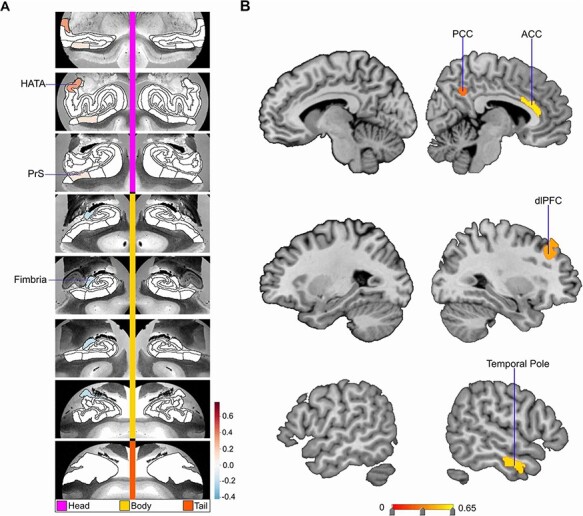
Structural divergences in left hemisphere HC subregions and right DN subregions are associated with low social support. Mode 4 of the CCA solution achieves the fourth most explanatory HC–DN co-variation signature, with a canonical correlation of rho = 0.31. (A) The figure shows the HC subregion patterns (left, one canonical vector of mode 4) with parameter weights that robustly diverge between low and high social support groups—mapped onto eight consecutive coronal slices of the left and right HC in the anterior (top) to posterior (bottom) direction. (B) The figure shows the DN subregion patterns (right, other canonical vector of mode 4) that robustly diverge between the low and high social support groups. These results further demonstrate that low social support is associated with distinct subregion divergences within-HC–DN co-variation signatures, which are robustly present in only one brain hemisphere.

**Fig. 4.**
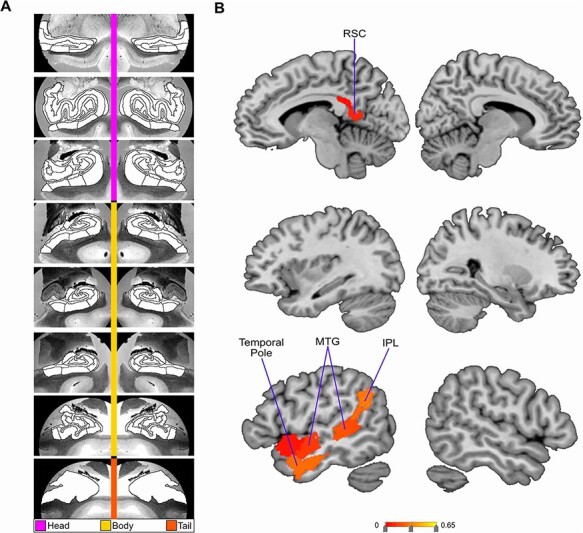
Low social support is associated with selective divergences in left lateralized DN subregion volumes. Mode 6 of the CCA solution acheives the sixth most explanatory HC–DN co-variation, with a canonical correlation of rho = 0.23. (A) The figure shows the HC subregion patterns (left, one canonical vector of mode 6) with parameter weights that robustly diverge between low and high social support groups—mapped onto eight consecutive coronal slices of the left and right HC in the anterior (top) to posterior (bottom) direction. (B) The figure shows the DN subregions patterns (right, other canonical vector of mode 6) that robustly diverge between the low and high social support groups. These results emphasize the selectivity of the social support and group difference analysis, as even though the analysis determines divergences on an individual subregion level, a coherent and highly lateralized set of subregions are highlighted.

**Fig. 5.**
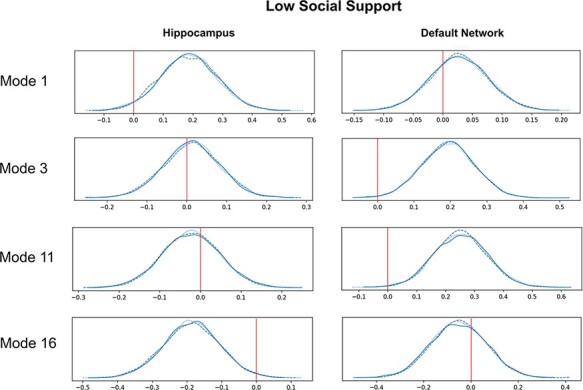
The genetic predisposition for low social support is linked to the brain signature expressions of HC–DN co-variation. We conducted a PRS analysis to estimate the subject-specific heritable tendency for low social support based exclusively on genome-wide effects across tens of thousands of SNPs. The subject-specific PRS estimates were then regressed against the expressions of each of the signatures of structural co-variation between the HC and DN. The relevance of the heritability effects was judged based on the posterior parameter distributions inferred by the Bayesian logistic regression model (histograms). The *x* axis of each plot represents the posterior parameter distribution for the β coefficient of the model, and the *y* axis of each plot represents the plausibility of each coefficient value. A value of 0 indicates no association between PRS and inter-individual mode expression. In each panel, the three posterior distribution histograms show convergence across three different Markov chain Monte Carlo runs (blue lines). Overall, heritability effects for low social support are related to the HC patterns of modes 1 and 16, as well as to the DN patterns for modes 3 and 11. These results demonstrate that specific mode expressions are robustly linked to the predisposition for lacking social support based on genetic complexion. The more explanatory modes with associations to heritability also show numerous subregion divergences in individuals with low social support (see Figures 1 and 2). In sum, structural divergences within specific HC and DN subregion co-variance patterns may be explained by the genetic predisposition for objective social isolation.

In the second most explanatory signature of HC–DN co-variation (i.e. mode 2), we identified seven HC hits—bilateral tail, bilateral parasubiculum, bilateral PrS head and left Sub body—and no hits in DN subregions (see Supplementary Figure S3). In mode 3, we observed two HC hits located in left CA2/3 head and right Sub head. We also observed seven DN hits, located in the ventromedial prefrontal cortex (vmPFC), dorsolateral prefrontal cortex (dlPFC) and TPJ—all of which were in the left hemisphere (see Figure 3). In mode 4, we observed three HC hits located in left PrS head, left fimbria and left HATA. All these HC hits for mode 4 were in the left hemisphere. Conversely, we observed four DN hits exclusively in the right hemisphere (see Figure 4). These DN hits emerged in the temporal pole, retrosplenial cortex (RSC), anterior cingulate cortex (ACC) and dlPFC. In mode 5, we observed four HC hits located only in the right hemisphere. These hits included the fimbria, PrS head, Sub head and molecular layer head. We also noted one hit in the DN: right posterior cingulate cortex (PCC) (see Supplementary Figure S4). In mode 6, we observed no HC hits and 12 DN hits all in the left hemisphere (see Figure 5). These hits included eight in the lateral temporal lobe, two in the IPL and two in the RSC. In mode 7, we observed a hit in the left parasubiculum and no DN hits (see Supplementary Figure S5). Beyond mode 7, we did not observe hits in any of the other 25 examined modes. Thus, the more dominant signatures of HC–DN co-variation showed stronger relationships to a lack of social support than less dominant signatures. We present here the modes with the greatest number of subregion divergences in both the HC and DN (see Figures 2–5). These collective results show that a group contrast analysis of low *vs* high social support individuals revealed specific subregion divergences within spatially overlapping signatures of HC–DN co-variation.

Overall, we noted a recurring theme of certain subregions with numerous hits in the group analysis of social support. For the HC, this included the PrS, Sub, CA2/3 and GC-DG-ML (see Table 1 for full list). For the DN, especially lateral subregions—such as the dlPFC, IPL and MTG—tended to diverge between low *vs* high social support groups (see Table 2 for full list). We also noted that the divergences observed for low social support occurred in structural patterns within each mode, as most hits were chiefly seen on only one brain hemisphere. For example, in mode 6, there was a cluster of hits in lateral cortical regions but only in the left hemisphere of the brain (see Figure 5). Analogously, in mode 3, we observed seven DN hits, yet all in the left hemisphere. Thus, low social support was primarily concomitant with divergences in left lateral DN subregions, as well as the PrS, Sub, CA2/3 and the GC-DG-ML of the HC.

**Table 2.** Default network subregion divergences across hippocampus–default network co-variation modes

Mode	Temporal	Prefrontal	Parietal	Posterior cingulate
1	8	7	10	1
2	0	0	0	0
3	0	5	2	0
4	1	2	0	1
5	0	0	0	1
6	8	0	2	2
Total	17	14	14	5

There were no subregion divergences between modes 7 and 25.

### Genetic predisposition for social isolation

**Fig. 6.**
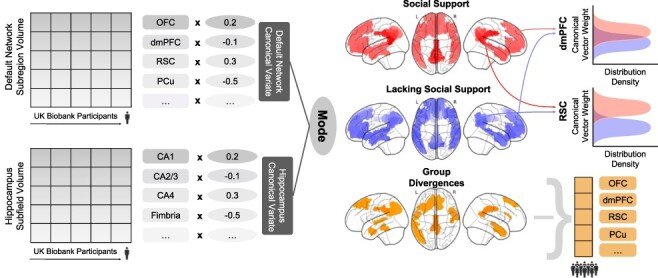
Workflow schematic to model differences in co-variation between HC subregions and DN subregions in individuals lacking social support. Dominant regimes of HC and DN co-variation were estimated at population scale through the CCA algorithm. Subsequently, the divergence of these patterns was determined by comparing low social support *vs* adequate social support groups in our UK Biobank cohort (*n* = 38 701). In particular, CCA allowed the joint investigation of the multivariate patterns of co-variance between the 38 HC subregions and 91 DN atlas subregions of the examined UK Biobank participants. The ensuing insight from CCA takes the form of ‘modes’ or signatures of co-variation, which emphasize the effect sizes of every HC and DN subregion in each identified mode. The identified pair of canonical vectors (for each mode) determines the maximally explanatory combination of weights which account for joint HC–DN structural co-variation throughout the UK Biobank cohort. The canonical vectors (i.e. modes 2–25) capture remaining variance in the brain-imaging data that is linearly independent of the previous modes’ explained variance. A robust difference for a particular subregion between individuals lacking social support and those with adequate social support is shown on the far right. The density distribution of canonical vector weights is shown among the aggregated 100 bootstrap datasets. The determination of a ‘hit’ (or a robust divergence in subregion volume) was determined based on whether the two-sided confidence interval included zero according to the 10/90% population-level certainty intervals (bootstrap) of the structural difference estimates.

We finally sought to interrogate whether the uncovered expressions of HC–DN co-variation featured systemic relationships with the participants’ liability for low social support (cf. ‘Materials and methods’ section). For this purpose, we computed PRS predictions for the innate risk of low social support for our UK Biobank participants. We observed that there was a relevant relationship between low social support PRS and participant expressions (i.e. canonical variates) of modes 1 and 16 for the HC and modes 3 and 11 for the DN (see Figure 6). The HC–DN modes with robust relationships were thus the relatively more dominant signatures among the 25 examined. The HC and DN patterns with relationships to PRS for low social support were also in modes in which we observed structural divergences in individuals with low social support. For example, mode 1 on the HC side showed 15 subregion hits (see Figure 2), and mode 3 on the DN side showed 7 subregion hits (see Figure 3). Overall, we found that specific mode expressions in low social support individuals had robust ties to purely genetic factors, as captured by genome-wide risk predictions across tens of thousands of genetic markers.

## Discussion

We have tailored an analytical framework to examine how the anatomical substrates of HC–DN correspondence systematically deviate in individuals lacking regular social exchange with close others. Our approach allows extending the emerging interpretations of distinct subsystems within the DN by establishing robust cross-links with anatomically defined HC subfields at the population level. We work toward this goal by directly estimating principled co-variation signatures that delineate how HC subregion volumes are co-expressed with DN subregion volumes in ∼40 000 participants from the UK Biobank imaging-genetics cohort. In so doing, our study aimed to deepen the understanding of the human DN by anchoring its variation in corresponding substrates of the allocortical HC—a structure that has been implicated in mnemonic and associative processes in non-human animals and which likely underpins human social navigation.

Past literature supports the notion that the frequency with which an individual interacts with other people resonates with the structural characteristics of the HC and DN. In fact, DN subregions are implicated in representations of oneself and social others (Mars *et al.*, 2012; Laurita *et al.*, 2020). Likewise, the human HC has been proposed to represent information about individual people (Quiroga *et al.*, 2005), in addition to its classic functional interpretations of representing places and retrieving memories (Scoville and Milner, 1957; O’Keefe and Dostrovsky, 1971). Previous authors have even argued that the HC may mediate an abstract social cognitive map for relationships between people, in the form of ‘vector angle’ representations (Tavares *et al.*, 2015). In light of this idea, we expected individuals with lower frequency of social interactions, or low social support, to exhibit differences in specific signatures of HC–DN co-variation, compared to individuals with higher levels of social connection and support. Although we are unable to establish whether the divergences in HC–DN co-variation are specific to social processing and exclude other cognitive operations subserved by this relationship, we show that our measure of social support frequency is associated with robust divergences.

In accord with our expectations, we identified a set of subregional divergences that were characteristic for individuals with low social support: PrS, Sub, CA2/3, GC-DG-ML and CA4 of the HC. In particular, the expressions of structural co-variation across all modes revealed a total of five PrS hits, five Sub, four CA2/3, four GC-DG-ML and three in CA4 for individuals who lack social interaction in everyday life. Effects in these five subregions alone constituted as much as 66% of the total observed subregion divergences. Experimental animal models of subregional specialization within the HC suggest that this may reflect differences in HC subfields, which represent discrete elements of social information (Watarai *et al.*, 2021). Such divergences would be in accord with the relationship between an individual’s objective level of social embeddedness and the richness of their neural representations of social information (Li *et al.*, 2013; Chadwick *et al.*, 2014; Watarai *et al.*, 2021).

More specifically, the CA2 subregion—which yielded four total hits across modes in the combined CA2/3 subregion—has been shown to be integral for successful peer recognition in rodents (Hitti and Siegelbaum, 2014; Stevenson and Caldwell, 2014; Alexander *et al.*, 2016; Leroy *et al.*, 2018; Meira *et al.*, 2018). For example, in mice, functional inhibition of dorsal CA2 cells through viral neurotoxin injection has been found to reduce interaction time with familiar peers (Hitti and Siegelbaum, 2014). Yet, this direct intervention on HC tissue did not appear to affect sociability or other HC-dependent behaviors (Hitti and Siegelbaum, 2014). Similarly, it has been reported that mice showed an altered rate of place cell remapping in CA2 after the introduction of a peer into their environment. Conversely, exposure to a novel toy, as an enrichment of the physical environment, did not produce such effects (Alexander *et al.*, 2016). In our study, the CA2 subregion was only analyzable through the combined CA2/CA3 subregion in the UK Biobank reference atlas because of the CA2 subregions’ small size and challenging brain-imaging-based demarcation. The combined CA2/3 subregion may thus offer a small window into the social memory functions of the HC. However, a limitation of this interpretation is that past authors have noted subregions with smaller anatomical size yield relatively less reliable measurements (Worker *et al.*, 2018; Quattrini *et al.*, 2020).

Individual cells in the dorsal CA1 have been found to support a similar function as CA2 cells, as CA1 cells have been reported to reliably represent the spatial location of peers (Danjo *et al.*, 2018; Omer *et al.*, 2018); however, CA2/3 cells were not recorded in these studies. Evidence that CA2 neurons especially encode information of newly encountered peers and possess place fields sensitive to social information has also recently been reported (Oliva *et al.*, 2020). Numerous studies across species have additionally suggested that neurogenesis—a process localized to the granule cell layer of the dentate gyrus—is particularly sensitive to the experience of social isolation (Stranahan *et al.*, 2006; Ibi *et al.*, 2008; Dranovsky *et al.*, 2011; Biggio *et al.*, 2019) and social stress (Gould *et al.*, 1997, 1998; Anacker *et al.*, 2018). Hippocampal neurons thus appear to play a committed role in instantiating and integrating representations of social agents. Our results are consistent with this idea, as we find preferential hits in CA2/3 and DG, indicating systematic differences in the neural representations of social information. Neural activity and structure of the human DG and CA2/3 may thus reverberate with the richness of the environment more broadly, including social agents and relationships between them.

Similarly, CA2/3 and the DG have been discussed to be instrumental in disambiguating sensory inputs from similar stimuli (Kesner and Rolls, 2015; Knierim and Neunuebel, 2016). This is a function that has been termed ‘pattern separation’ and has been established at a single-cell level in the rodent HC (Gilbert *et al.*, 2001; Leutgeb *et al.*, 2004, 2007) and through neuroimaging in humans (McHugh *et al.*, 2007; Bakker *et al.*, 2008; Baker *et al.*, 2016; Berron *et al.*, 2016). An emerging view is that pattern separation is a key function altered in AD (Ally *et al.*, 2013; Wesnes *et al.*, 2014; Parizkova *et al.*, 2020), while AD has analogously been linked with social isolation (Marioni *et al.*, 2015; Penninkilampi *et al.*, 2018; Shen *et al.*, 2021; Xiang *et al.*, 2021). For example, in a mouse model of AD, the investigators reported reduced Aβ synaptotoxicity in the HC (Li *et al.*, 2013) and reduced cognitive impairment (Jankowsky *et al.*, 2005) with environmental enrichment—an important source of which in humans is regular social contact. Recently, invasive cell-recording experiments also showed that AD model mice have a selective impairment in pattern separation as subserved by the DG and CA3 (Rechnitz *et al.*, 2021). Overall, the neurobiological underpinnings for representing a rich environment, perhaps best constituted by mapping social networks with their ties among people, may be intimately linked with AD and its accompanying cognitive consequences. Our study speaks to an association of divergences in the DG and CA2/3 with objective social isolation. This population-level insight highlights these allocortical subregions and their neocortical affiliates as important targets for future investigations on pattern separation-dependent functions and AD.

Of course, neural representation of social information and social processing are not the sole provenance of HC subregions. Rather they involve dynamic functional coupling with the neocortex to support social labeling and the construction of more complex models of the social environment (Schurz *et al*., 2021). Our analytical approach was designed to directly investigate this aspect of allocortical–neocortical correspondence in the service of social functioning. Our results underscore structural divergences in left lateral temporal and lateral parietal subregions of the highly associative DN in individuals with low social support—neocortical divergences that our approach revealed to be concomitant with the allocortical divergences in CA2/3 and DG. We speculate that these conjoint divergences in left lateral temporal and parietal subregions, which are implicated in social semantics and spatial processing respectively (Mars *et al.*, 2012; Alcalá-López *et al.*, 2018; Hartwigsen *et al.*, 2021), are linked to the previously reported functional roles served by CA2/3 and DG in social cognition. This includes roles such as binding information about social relationships within a social cognitive map.

Further, we observed subregion divergences in individuals with objective social isolation to occur in almost exclusively lateralized patterns. These were consistent with left-lateralized semantic processes (Binder *et al.*, 2009; Liu *et al.*, 2009; Numssen *et al.*, 2021). Indeed, the vast majority of our observed DN hits were in left-hemispheric subregions in modes 1, 3 and 6 (76% of the total), consistent with the idea that sociality is fundamentally dependent on semantics as well as conceptual and symbolic (e.g. language) processing (Dunbar and Shultz, 2007; Frith and Frith, 2010; Lord, 2013). For example, much of the everyday stimulation in people’s lives is driven by social information (Mesoudi *et al.*, 2006; Mar *et al.*, 2012). Low access to social exchanges with other people may therefore be appropriately viewed as a condition for an overall stimulation-poor environment. Indeed, several past studies have made evident that our highlighted DN subregions are neurobiological substrates of environmental vibrancy. As some of many examples, volume and density of temporal lobe GM—especially the MTG and superior temporal sulcus—have been shown to track social network size, both as measured by online interactions in humans (Kanai *et al.*, 2012), as well as in real-world social groups of monkeys (Sallet *et al.*, 2011).

The anterior portion of the human HC has also been proposed to be a locus of both semantic (Schacter and Wagner, 1999; Strange *et al.*, 2014) and social (Vogel *et al.*, 2020; Morton *et al.*, 2021) processing. In line with this notion, we identified 21 total hits in the head portion of the HC (66%) and only 11 (34%) in its body portion across all modes. The frequency of meaningful encounters with close others thus appears to be associated with divergences of structural co-variance signatures involving the anterior HC. When considered in conjunction with the preferential structural divergences of left-lateralized DN subregions, these two broad patterns reconcile previous reports of sub-specializations within each of these two brain systems (Andrews-Hanna *et al.*, 2010; Fanselow and Dong, 2010). In sum, social cognition draws upon social concept representation and abstract cognitive maps among other processes, which are revealed to be subserved by the concord of lateral subregions of the DN with CA2/3, DG and the broader anterior HC.

The DN has also been reliably dissociated into subsystems (Andrews-Hanna *et al.*, 2010; Braga and Buckner, 2017; Braga *et al.*, 2019, 2020; DiNicola *et al.*, 2020). Our population neuroscience results confirm and detail this notion by uncovering distinct signatures of structural deviation in social isolation that aligns well with one of these subsystems. More specifically, our results implicate structural deviations in lateral DN subregions in objective social isolation, while past studies have highlighted together the lateral temporal cortex, TPJ, dmPFC and temporal pole. Nonetheless, our study goes beyond previous attempts to sub-divide the DN, as we extend the interpretational themes of each DN subregional pattern by appreciating, and explicitly modeling, its consistent structural relationships with dedicated hippocampal subregions. In particular, the hippocampal patterns robustly linked with lateral temporal and parietal subregions tended to highlight CA2/3, GC-DG-ML and CA4. Overall, these observations reinforce the idea of biologically coherent cross-dependencies that exist between these subregions of the DN and HC.

Since there appears to be an inherent relationship between objective social isolation and the HC–DN correspondence highlighted in our results, we suspected there may be a heritable contribution to these characteristics. To this end, we conducted the first—to our knowledge—PRS analysis for low social support. This analysis allowed us to investigate the genetic contributions to low social support that are due to individually small-effect size SNPs. Overall, we found that participant-specific expressions of HC–DN signatures show differing links to the heritable components of low social support. This is in accord with some previous research showing that social isolation has consistent, yet small genetic underpinnings (Spithoven *et al.*, 2019). However, the majority of past investigations of the genetics of social isolation have focused on the subjective feeling of social connection (i.e. loneliness) (Boomsma *et al.*, 2005; Gao *et al.*, 2017; Day *et al.*, 2018; Abdellaoui *et al.*, 2019).

Yet, an underlying genetic contribution to objective levels of social support has been supported by recent genome-wide correlation analyses. For example, one study has demonstrated that our UK Biobank social support trait significantly shared genetic factors with 52 different demographic, lifestyle and disease phenotypes (Schurz *et al.*, 2021b). Such recent population neuroscience research indicates that the genetic determinants underlying social isolation are probably quite polygenic and involve complex gene–environment interactions. In our present study, we extended these insights by performing a PRS analysis for low social support. We furthered this by elucidating the relationship between a participant-specific predisposition for lacking social support and the brain expression of each HC–DN co-variation signature. Importantly, we found that only selected signatures of HC–DN co-variation showed a relevant relationship with genetic liability for low social support. These precise modes additionally showed numerous subregion divergences in our group difference analysis, which roughly overlapped the subregion divergences observed in low social support (i.e. modes 1 and 3). For example, expression of mode 1 on the HC side was linked to PRS for low social support and highlighted the CA2/3 and DG subregions. Participant-specific expressions of mode 3 on the DN side were also linked with PRS for low social support and showed seven DN subregions with structural divergences, all in the left hemisphere. The genetic predisposition for low social support is thus manifest in brain networks that follow a pronounced left–right divide, reminiscent of the left-lateralized nature of semantic networks that subserve human-defining cognition (Binder *et al.*, 2009; Liu *et al.*, 2009; Numssen *et al.*, 2021).

However, the determination of ‘objective’ social isolation in our study is not without limitations. Past studies have shown that single-item phenotypes from the UK Biobank exhibit statistically robust genetic correlations (Spreng *et al.*, 2020; Schurz *et al.*, 2021b), even though the identification of participants with a lack of social support is based on a self-reported answer to a single question. Many authors view social support as a measure of objective social isolation (Cacioppo *et al.*, 2011, 2015; Taylor *et al.*, 2018), but a future protocol to more rigorously identify social support metrics is thus warranted. Notwithstanding this limitation of our study setup, our results identify definite structural brain pattern differences between well-connected participants and those deemed to objectively lack social support, which is sensible and plausible in terms of past literature.

In conclusion, enabled by the breadth and depth of the UK Biobank resource, our analytical approach showed that an objective measure of social connection has robust structural concomitants in human HC and DN subregions. These allocortical–neocortical structural deviations included PrS, Sub, CA2/3 and DG subregions of the HC and lateral subregions of the DN. Our framework extends understanding of the functional subsystems of the DN and their potential preferential relationship with dedicated HC subfields. We also found that distinct signatures of HC–DN structural co-variation have robust relationships with individuals’ genetic predisposition for social disconnection. Future investigations into the genetic determinants and environmental influences on social isolation represent important research directions to further elucidate the structurally heterogenous qualities of the socially isolated brain.

## Supplementary Material

nsac006_Supp
