# Supplementary material for: Lacking social support is associated with structural divergences in hippocampus–default network co-variation patterns
Source: Soc Cogn Affect Neurosci. 2022 Jan 27;17(9):802–18. doi: 10.1093/scan/nsac006 (PMC9433851; doi:10.1093/scan/nsac006)
Supplement: nsac006_Supp [file nsac006_supp.zip › scan-21-236-File009.docx]

**Supplementary Table 1: Sociodemographic characteristics**

| **UK Biobank ID** | **Description** | **Regular social support** | **Lack of social support** |
| --- | --- | --- | --- |
|  | Overall participants | 54.85% | 45.15% |
| 21022 | Age (years) | 54.73 (+/- 7.45 SD) | 55.03 (+/- 7.49 SD) |
| 31 | Sex (men : women) | 47.00% : 53.00% | 48.05% : 51.95% |
| 20016 | Fluid IQ (0=low, 13=high) | 6.66 (+/- 2.00 SD) | 6.74 (+/- 2.08 SD) |
| 2020 | Loneliness (often feels lonely) | 7.82% | 23.00% |
| 709 | Number of people living in household | 2.67 (+/- 1.16 SD) | 2.41 (+/- 1.27 SD) |
| 845 | Age completed school education (years) | 17.07 (+/- 2.74 SD) | 17.06 (+/- 2.84 SD) |
| 738 | Average total household income before tax (5 = high, 1 = low) | 3.11 (+/- 1.10 SD) | 2.82 (+/- 1.15 SD) |
| 6142 | Employment status (payed full time job = 1, not = 0) | 70.3% | 69.31% |
| 1239 | Current tobacco smoking (on most or all days) | 3% | 5% |
| 20403 | Amount of alcohol drunk on a typical drinking day | 2.20 (+/- 0.74 SD) | 2.23 (+/- 0.74 SD) |
| 1873 | Number of full brothers | 1.06 (+/- 1.14 SD) | 1.07 (+/- 1.15 SD) |
| 1883 | Number of full sisters | 0.98 (+/- 1.09 SD) | 1.00 (+/- 1.00 SD) |
